# A Soft-Inflatable Exosuit for Knee Rehabilitation: Assisting Swing Phase During Walking

**DOI:** 10.3389/frobt.2018.00044

**Published:** 2018-05-17

**Authors:** Saivimal Sridar, Zhi Qiao, Niveditha Muthukrishnan, Wenlong Zhang, Panagiotis Polygerinos

**Affiliations:** ^1^The Polytechnic School, Ira A. Fulton Schools of Engineering, Arizona State University, Mesa, AZ, United States; ^2^School for Engineering of Matter Transport and Energy, Ira A. Fulton Schools of Engineering, Arizona State University, Tempe, AZ, United States; ^3^School of Biological and Health Systems Engineering, Ira A. Fulton Schools of Engineering, Arizona State University, Tempe, AZ, United States

**Keywords:** soft-inflatable actuators, soft exosuit, stiffness control, wearable robotics, assistive devices

## Abstract

In this paper, we present a soft-inflatable exosuit to assist knee extension during gait training for stroke rehabilitation. The soft exosuit is designed to provide 25% of the knee moment required during the swing phase of the gait cycle and is integrated with inertial measurement units (IMUs) and *smart shoe* insole sensors to improve gait phase detection and controller design. The stiffness of the knee joint during level walking is computed using inverse dynamics. The soft-inflatable actuators, with an I cross-section, are mechanically characterized at varying angles to enable generation of the required stiffness outputs. A linear relation between the inflatable actuator stiffness and internal pressure as a function of the knee angle is obtained, and a two-layer stiffness controller is implemented to assist the knee joint by providing appropriate stiffness during the swing phase. Finally, to evaluate the ability of the exosuit to assist in swing motion, surface-electromyography (sEMG) sensors are placed on the three muscle groups of the quadriceps and two groups of the hamstrings, on three healthy participants. A reduction in muscle activity of the rectus femoris, vastus lateralis, and vastus medialis is observed, which demonstrates feasibility of operation and potential future usage of the soft inflatable exosuit by impaired users.

## 1. Introduction

Stroke, according to a NCBI study is the number one cause of chronic disability worldwide (Plummer et al., 2007) with about 6.6 million stroke survivors in the United States of America alone (Lai et al., 2002). About 30% of these survivors have difficulty in ambulation without assistance and 26% are unable to perform activities of daily living requiring physical therapy and rehabilitation. Stroke is known to cause paresis-weakness of muscles or plegia -complete loss of muscle action, in upper and/or lower limbs depending on the severity of the stroke episode and locus of brain damage. According to reports post-stroke, gait patterns often have deviations in kinetic, kinematic and spatiotemporal characteristics such as reduced walking speed, decreased ability to produce moment of force or decreased muscle power in quadriceps muscles, that are responsible to produce leg extension motion and maintain knee joint stability during walking (Hamrin et al., 1982; Harris et al., 2001; Thompson et al., 2013).

Increasing efforts for physical rehabilitation of paretic limbs have been made in the form of assistance using passive, quasi-passive and active devices to assist in knee extension (Pratt et al., 2004; Banala et al., 2006; Aubin et al., 2013). The two most important limitations associated with passive devices are that they provide inadequate assistance to alter abnormal gait pattern and cannot supply energy to the limb. To resolve the aforementioned, there has been extensive research on designing rigid exoskeletons and other quasi-passive or active devices with the aim to actively assist in rehabilitation therapy for the knee joint during walking (Aguirre-Ollinger et al., 2007; Roy et al., 2009; Bulea et al., 2017). Several types of hardware designs and software controllers have been implemented, including stiffness controllers, to provide appropriate assistance to the paretic limbs (Huo et al., 2015; el zahraa Wehbi et al., 2017). These active devices are capable of providing assistance and support in applications where larger forces are necessary (del-Ama et al., 2012). However, the primary concern with the use of some active devices, such as exoskeletons, is that they are heavy, bulky, expensive, not portable and may require a team of clinicians for supervised therapy sessions, which are often not reimbursed through health insurance (Godwin et al., 2011). Although, there have been several noteworthy advancements in rigid exoskeleton systems that successfully reduce their weight significantly, these rehabilitation devices, most of the time require precise alignment with the biological joints that without clinical supervision can induce additional issues on the currently impaired gait biomechanics. On the contrary, soft robotic devices, due to their lightweight and compliant nature, as well as inexpensive development cost, could potentially overcome these issues by reducing dependence on clinical resources (Polygerinos et al., 2017). Because of the additional unique traits of soft robots, such as high power-to-weight ratio and ease of manufacturing, they have demonstrated great promise in aiding therapy of stroke afflicted patients (Awad et al., 2017).

**Figure 1:**
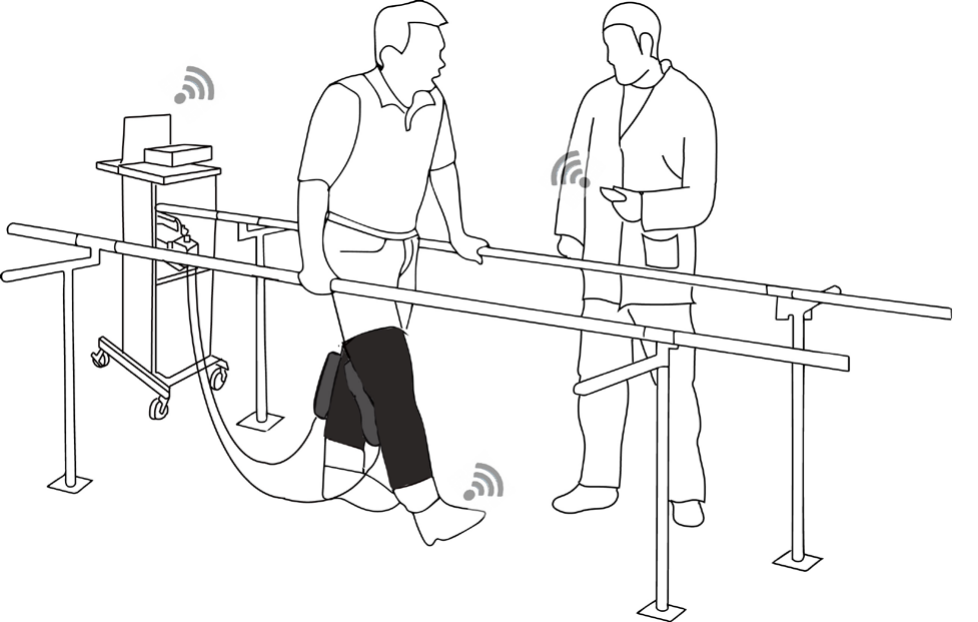
1 Illustration of the soft-inflatable exosuit and its utilization in a rehabilitation setting with the physical therapist wirelessly tuning the assistance provided by the exosuit to the user based on sensory outputs from the *smart shoes* and IMUs.

Building upon the advantages offered by soft robotics, in this paper we present a soft-inflatable exosuit that aims to assist the knee extension motion of the swing phase during gait rehabilitation. There have been previous research studies in assisting the stance phase using exoskeletons (Wong et al., 2012; Byl, 2012) but allowing free motion during the swing phase. Our main goal is to provide preliminary evidence to support the hypothesis that the soft inflatable exosuit design can effectively reduce muscle effort during knee swing motion in healthy users, thus opening the path for future assistance of individuals with lower-limb impairments. An illustration of the operation concept is shown in Figure 1, while the contributions of this work are outlined as follows:

- Wireless inertial measurement units (IMUs) and *smart shoe* insole sensors (Zhang et al., 2016), which can provide accurate estimation on the knee joint angles and ground reaction forces (GRF) in real-time, are integrated into the soft inflatable exosuit design (Sridar et al., 2017).
- A two-layer control algorithm with a high-level stiffness controller which takes inputs from the IMU and the *smart shoe* to generate a desired pressure reference signal for the low-level controller and a low-level pressure controller for tracking the pressure trajectory.
- A stiffness model for the I cross-section (Sridar et al., 2017) soft-inflatable actuators as a function of knee flexion angle and internal pressure that aids in the generation of the desired stiffness.
- Demonstration of the effectiveness of the soft inflatable exosuit through a healthy participant study that investigates the levels of muscle effort exerted with and without the device active.

 The remainder of the paper is organized in sections as follows: Section 2.1 discusses the design of the soft inflatable exosuit, off-board control unit and the knee angle and gait measurement system. The control system design and the stiffness modeling of the human knee joint as well as the soft inflatable actuators is presented in Section 2.2. Section 3 presents the pressure tracking experiment for the exosuit as well as the participant testing evaluation using sEMG and the results are discussed in Section 4. Finally, the conclusions and future work are stated in Section 5. 

## 2. Materials and Methods

### 2.1. System Design

#### 2.1.1. Soft-Inflatable Exosuit

**Figure 2:**
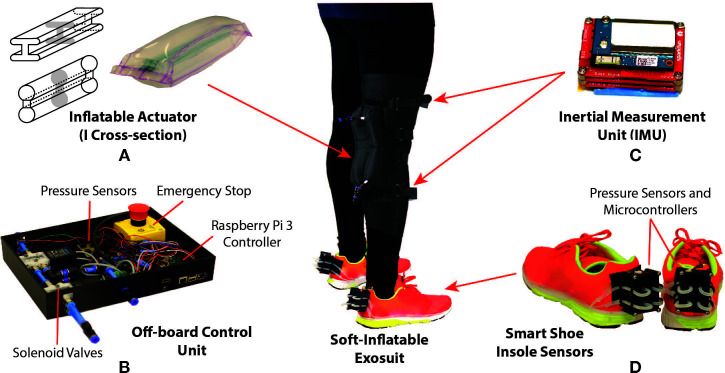
Design of the soft-inflatable exosuit **(A)** Inflatable actuators of I cross-section designed using heat sealable TPU material. **(B)** Off-board control unit containing solenoid valves, Raspberry Pi 3 controller, voltage regulators and emergency stop. **(C)** Inertial measurement unit mounted on the exosuit to measure knee angle. **(D)***Smart shoe* insole sensors to determine gait phases during walking.

In many paretic and hemi-paretic patients, the use of the quadriceps muscles is partially or completely lost. In cases with partial loss of muscle activity, the use of a rehabilitative device to compensate for the deficit is common practice. Our soft exosuit is designed to provide 25% of the peak torque generated by a healthy male human during the swing phase of walking. As described in our previous work, which introduces the novel design of our system (Sridar et al., 2017), the soft exosuit consists of two inflatable soft actuators with an I cross-section fabricated using thin thermoplastic urethane (TPU) films, shown in Figure 2A. The exosuit sleeve is designed using elastic fabric (neoprene), hence achieving maximum body conformity and transparency to the wearer. The inflatable actuators are encapsulated in inelastic fabric pockets sewn onto the elastic sleeve. These pockets are sewn such that the inflatable actuators make an equal lever arm about the knee joint which provides maximum assistance when inflated. Hook and loop straps are also attached to the elastic sleeve allowing for adjustable fit and comfort. Besides the adjustability, the hook and loop straps also aid in the uniform distribution of the generated forces to the thigh and the calf, thus providing maximum torque transfer to the body. Compared to our previous design, several critical improvements have been incorporated to aid the controlled operation of the exosuit, such as increasing the size of fluidic channels interconnecting the sections of the inflatable actuator to facilitate faster inflation and deflation rates, and direct attachment of pressure sensors to the inflatable actuators to increase accuracy of the pressure feedback for better control.

#### 2.1.2. Off-Board Control Unit

To reduce the weight of the exosuit, the pneumatic system and the electro-mechanical components required for the exosuit are stowed in an off-board setup. As shown in Figure 2B, the control unit utilizes off-the-shelf components to provide actuation for the exosuit. The control unit is equipped with high-speed/high-flow solenoid valves (MHE3-MS1H valves, Festo, Hauppauge, NY) which can operate at a maximum frequency of 200 Hz with a volume flow rate of 100 *L/min*. The valves are controlled using a micro-controller (Raspberry Pi 3, Raspberry Pi Foundation, United Kingdom), integrated pressure sensors (ASDXAVX100PGAA5, Honeywell International Inc., Morris Plains, NJ) to measure the pressure inside the inflatable actuators, and voltage regulators are incorporated to provide appropriate power supply to the components. Additionally, an external air compressor is used to provide positive pressure air supply to the actuators while and a vacuum pump (DV-85N-250 pump, JB Industries, Aurora, IL) is connected to the exhaust valves to assist with the deflation of the soft actuators. The vacuum pump allows for the rapid deflation of the actuators during certain phases of the gait cycle.

An external AC supply is used to power the control unit along with all the components. Quick connect fittings are added to the system to attach and detach the exosuit to the control unit with ease also allowing for swift transportation of all the components if required. An emergency stop switch is also incorporated to provide a fail-safe mechanism for the electro-pneumatic components and protect the end user, as required by the institutional review board (IRB).

#### 2.1.3. Knee Angle and Gait Measuring System

In this work, we also introduce to the exosuit, two IMUs (LSM9DS0) and a wireless gait monitoring system, referred to as *smart shoes* (Zhang et al., 2016). These nine degree-of-freedom (DOF) IMU sensors are composed of a three DOF gyroscope, a three DOF accelerometer, and a three DOF magnetometer. Both accelerometer and gyroscope readings are commonly used to estimate the rotation angle. However, the accelerometer and gyroscope readings are prone to high frequency noise and drift respectively. Therefore, to improve the accuracy of knee joint angle estimation, a gradient descent filter (Madgwick et al., 2011) is applied to IMU raw data. To estimate the knee joint angle, the IMUs are placed in parallel with the femur and tibia, shown in Figure 2C. After aligning the sensors’ coordinate frames, the knee joint angle is calculated by comparing the roll angles between the two IMUs. The roll angle estimation is sampled at 100 *Hz* and broadcasted through a secure wireless ad-hoc network, which utilizes dedicated slave (Intel Edison, Intel, USA) and the Raspberry Pi 3 (Raspi 3) master micro-controllers.

**Figure 3:**
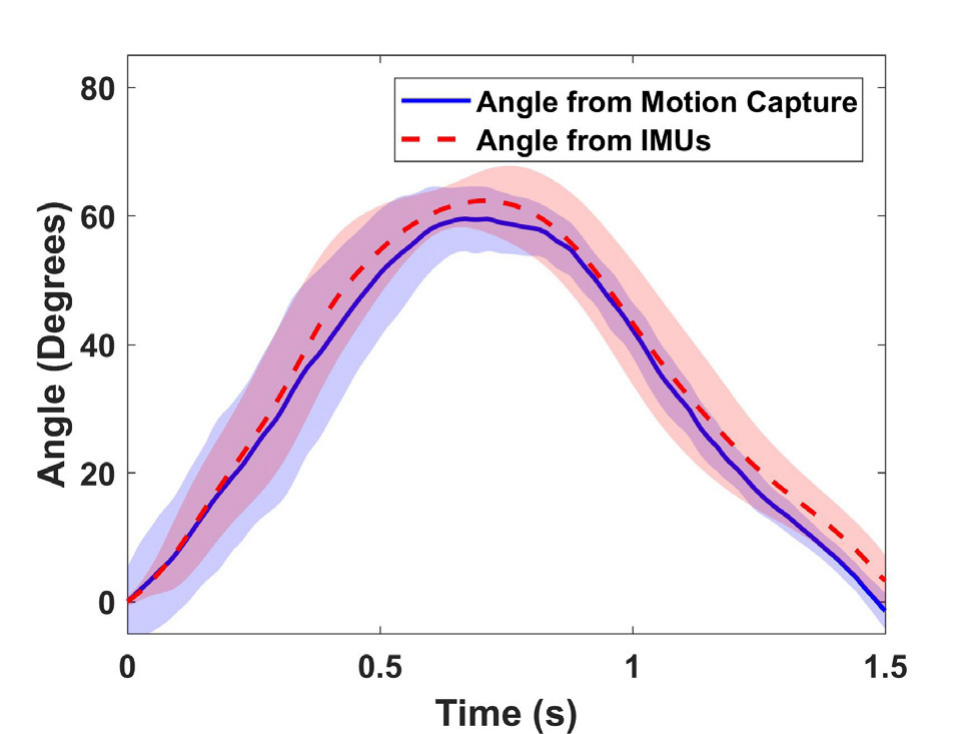
Joint angle measured by IMU (deg) against measurement from motion capture (deg) averaged for five cycles of operation with standard deviation (RMSE = 0.19*º* and error = 0.32%).

To determine the accuracy of the knee angle measurement system, the IMUs are mounted onto the tibia and femur of a 3D printed artificial knee joint fabricated using acrylonitrile butadiene styrene (ABS) plastic (Fortus 450mc, Stratasys, Eden Prairie, MN).Passive reflective markers are placed on the femur, knee joint, and tibia of the analog leg and a motion capture system (T40s, VICON Inc., Los Angeles, CA.) is utilized to provide the ground truth for the joint angle measurement. The results obtained from both the motion capture system and the IMUs are compared to verify the accuracy of the angles measured by the IMUs. As seen in Figure 3, the knee angle measured with the IMUs accurately tracks the knee angle obtained using the VICON system, with an RMSE of 0.19*º*. It is noted that when compared to traditional knee joint angle measuring systems (Karavas et al., 2013) with encoders and rigid braces, an IMU based system allows for free motion of the joints and provides the angle estimation in a lightweight setup with partially reduced measurement accuracy. Also, the use of encoders and other rigid measurement apparatus are not feasible for our system since we sacrifice the soft nature of the exosuit.

To provide estimates of the gait events during walking, the *smart shoe* insole sensor is integrated into the soft exosuit. The insole sensor contains four coils made of silicone tubing, attached to the bottom of a shoe sole in order to provide contact at four different points on the feet (i.e., heel, toe, the first (Meta1) and fourth (Meta4) metatarsal joint). Each of the coils is sealed on one end and the other end is connected to an air pressure sensor which reads the change in pressure when the coil is compressed. After calibration, the pressure variation is mapped into GRF changes. Similar to the IMUs, the GRFs are recorded and broadcasted to the same ad-hoc wireless network. Compared to force sensitive resistors (FSR) (Bamberg et al., 2008; Hanlon and Anderson, 2009), the *smart shoe* are accurate and stable over a long duration of use, eliminating noise and drift observed in FSR based systems (Chinimilli et al., 2016). With the GRF measurement from *smart shoe*, the gait events can be identified using fuzzy logic (Zhang et al., 2016). To decrease the time spent on fuzzy logic calculation, we introduce a simplified gait event detection rule. The output of the sensing point on heel and Meta4 is defined in two states: high and low. When the pressure measurement on heel or Meta4 is over 15% of the participant’s weight, both the sensing points will create a high logic output. By combining logic outputs from the two sensing points, different gait events such as heel strike (HS), foot flat (FF) and toe off (TO) can be successfully identified.

### 2.2. Control System Design

#### 2.2.1. Control System Overview

**Figure 4:**
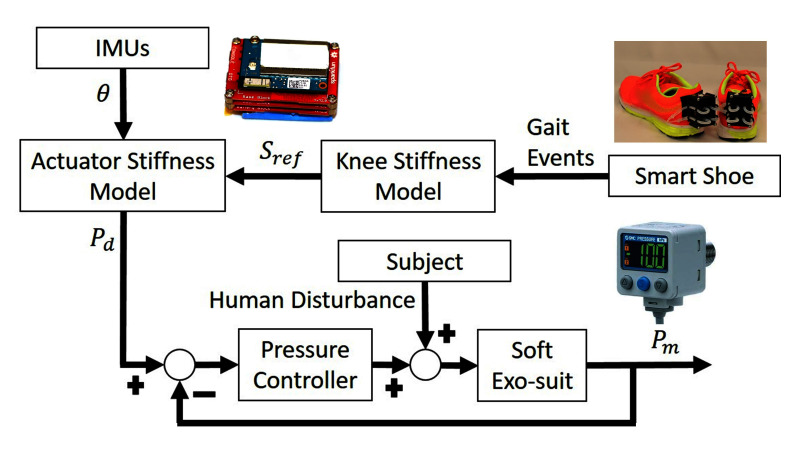
Control scheme of the soft exosuit. Measurements from IMUs and smart shoe are provided as an input to the actuator stiffness model to generate desired pressure set points. A low level pressure control loop is designed to drive the actuator to the pressure set points.

A two-layer control algorithm is proposed to tune the actuator stiffness and provide appropriate physical assistance based on the human gait events, as shown in Figure 4.

The first layer is a high-level controller, composed of the *smart shoes*, the knee stiffness model, the IMUs, and the actuator stiffness model. This layer takes measurements from the *smart shoes* and IMUs as inputs and generates a pressure reference to provide a 25% physical assistance at all times during the swing phase. The *smart shoes* provide the GRF estimation to detect gait phases in real time. As mentioned in (Karavas et al., 2013), the human knee stiffness value varies in different gait phases. With the gait information provided by *smart shoe*, the stiffness reference of soft exosuit $S_{ref}$ is calculated through the knee stiffness model. Taking the stiffness reference and knee angle θ measured by IMUs as inputs, the desired pressure value $P_{d}$ is calculated using the actuator stiffness model and treated as the final output of the high-level controller.

The second layer is a low-level pressure controller, which consists of the electro-pneumatics and the inflatable actuators. The low-level controller is a closed-loop system which utilizes the feedback from the pressure sensor connected to the inflatable actuators, to attain the desired pressure defined by the high-level controller at every instant. As mentioned in our previous work (Sridar et al., 2017), the binary control algorithm is used to control the actuator reach the desired pressure.

#### 2.2.2. Modeling of Knee Stiffness

**Figure 5:**
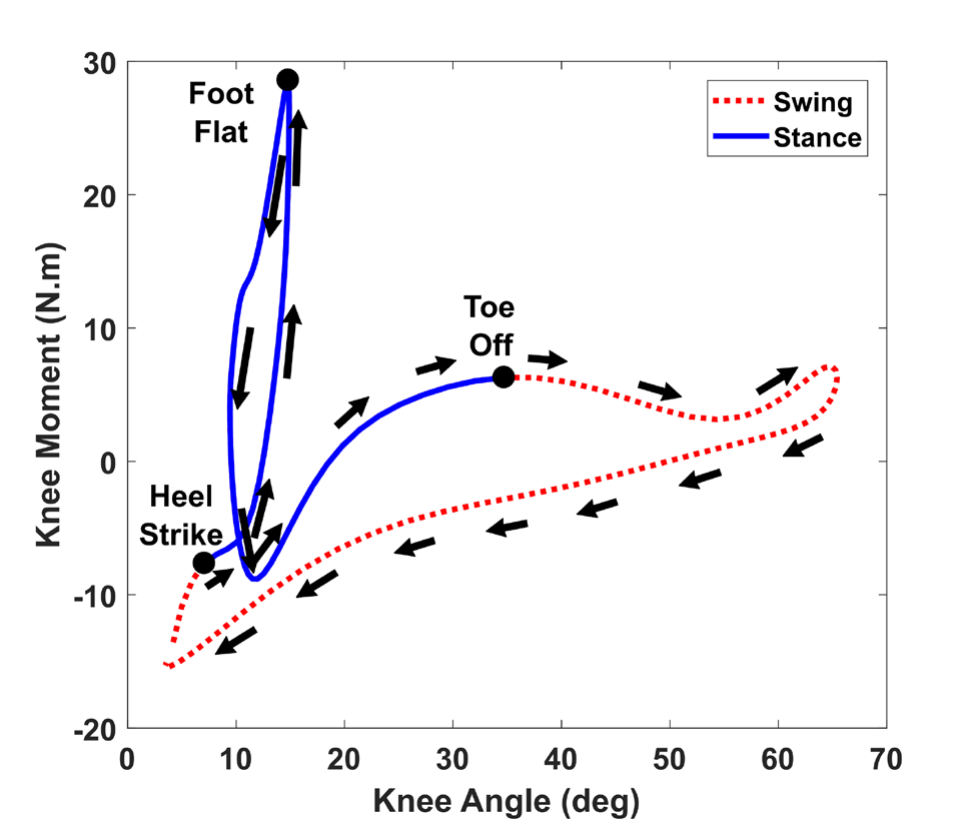
Modeling of human knee stiffness during walking. The averaged knee stiffness for stance and swing phase are calculated individually using the slope of the curve at the respective phase.

An experimental study was performed to model the knee joint stiffness of a healthy human during walking, using the VICON system and an instrumented treadmill (Bertec Corp., Columbus, OH.). The knee angles and GRF were collected during walking and the knee torque was calculated based on inverse dynamic algorithms, provided by the VICON plug-in gait model. Each gait cycle was divided into two phases: stance and swing. The stance phase was identified with the detection of HS, ending at the TO while the remaining of the gait cycle was defined as the swing phase. The relationship between knee joint toque and angle is shown in Figure 5. With the assumption that the knee joint can be modeled as a spring-damper system, the relationship between the knee moment and knee angle can be described as (Kearney and Hunter, 1990): 

(1) $$T\left(s\right)=\left(Is^{2}+Bs+K\right)\theta \left(s\right)$$

where, T is the torque, θ is the angle, I is the inertia, B is the viscosity and K is the stiffness of knee joint. Since the participant only walked at low-speed, the influence of the inertia and viscosity term were not significant. Moreover, the measurements of angular velocity and acceleration can be noisy, which make the inertia and viscosity terms less applicable in the model. Since this paper does not focus on providing assistance during stance phase, only the stiffness value during the swing phase was calculated using MATLAB 2016b System Identification Toolbox. The averaged swing knee stiffness values for a healthy subject walking at 0.5 *m/s* were determined to be 1.07 *N*⋅*m/deg*.

#### 2.2.3. Data-Driven Modeling of Inflatable Actuators

**Figure 6:**
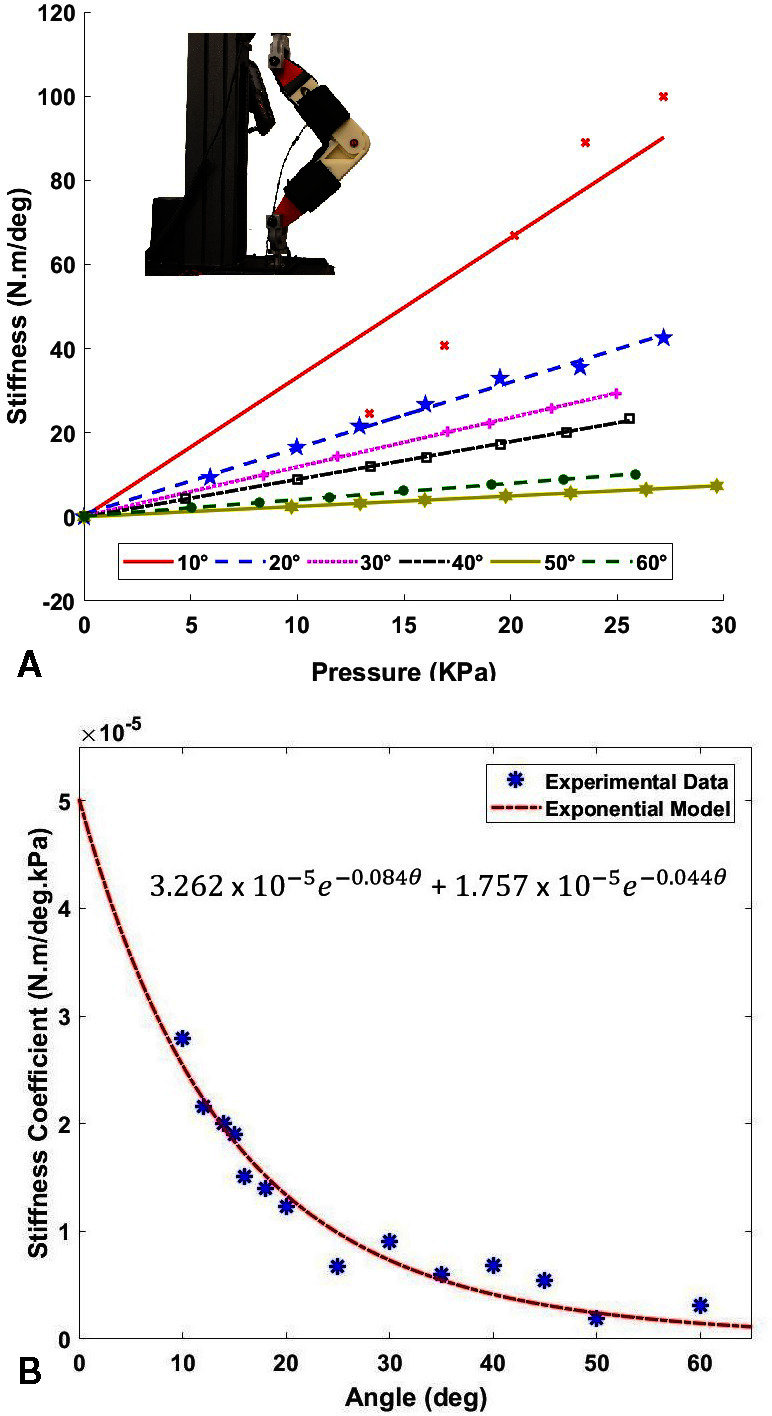
**(A)** Pressure (*kPa*) vs Stiffness (N.m/deg) of the inflatable actuators at varying angles. Testing setup mounted on a universal testing machine is shown in the inset. **(B)** Coefficient of stiffness K(θ) (*N.m/deg.kPa*) plotted against the flexion angle θ(deg). The equation of the stiffness coefficient K(θ) as a function of angle θ is shown.

A similar testing process as described in (Sridar et al., 2017) was followed to model the relationship between stiffness output and bending angle of the inflatable actuators at varying pressures. To measure the force output from the inflatable actuators in quasi-static condition, the artificial knee joint was mounted securely on a universal tensile testing machine equipped with a load cell (Instron 5944, Instron Corp., High Wycombe, United Kingdom) to capture the force data as shown in the inset of Figure 6A. The flexion angle of the knee joint was varied from 10*º* to 60*º* keeping it consistent with the flexion angle of the human knee during the gait cycle. The relation between pressure and stiffness output was computed at every 10*º* of the bending angle by varying the pressure from 0 to 27.58 *kPa* in intervals of 3.45 *kPa* to model the stiffness output of the inflatable actuators against the bending angle varying pressures. As seen in Figure 6A, for a fixed angle,the relationship between pressure sand stiffness appeared to be linear. Therefore, to compute the stiffness of the inflatable actuator as a function of internal pressure, it was hypothesized that there exists a relation such that

(2) S=K(θ)⋅P

where, S is the actuator stiffness, θ is the bending angle, and K(θ) is the actuator stiffness coefficient as a function of the angle. Therefore, to determine K(θ), as a function of the angle θ, the slopes of the stiffness at the corresponding angle were derived and plotted against the angle, as shown in Figure 6B. An exponential model was fit to the data using a non-linear least squares method, and the soft inflatable actuator stiffness coefficient was determined, shown in Figure 6B. The exponential fit (r-squared value of 0.95) was found to account for the high decline in the stiffness coefficient at low angles, and low decline during higher values of the knee flexion angle.

## 3. Results

### 3.1. Pressure Tracking

**Figure 7:**
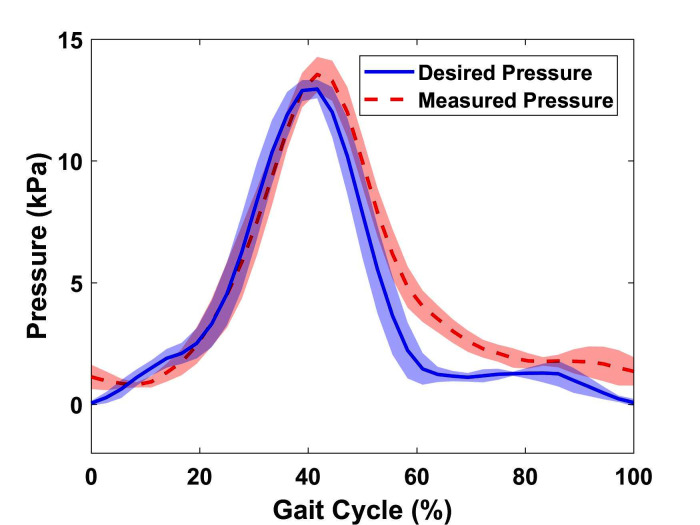
Desired pressure calculated by algorithm (*kPa*) vs. Actual pressed measured by the pressure sensor (*kPa*) over five gait cycles with SD. (RMSE = 1.27 *kPa* and error = 8.46%)

To quantify the performance of the low-level pressure control of the soft exosuit, the desired pressure calculated by the high-level control system and the actual pressure measured by the pressure sensor are compared, as shown in Figure 7. It is observed that during inflation, the actuator closely follows the desired pressure profile while during deflation, there exists a delay. This is because the flow rate is directly proportional to the pressure gradient leading to lower exhaust rates at lower pressures. Other contributing factors may include the constant suction rate of the vacuum pump in combination with the small in diameter pneumatic exhaust ports and lines. In addition to the slow deflation speed, a pressure overshoot at the maximum desired pressure is observed in all cycles. Possible explanation involves the non-optimal binary control algorithm parameters. Although there are overshoots at peak points and a delay in deflation, the RMSE value for the entire experiment is only 1.27 *kPa*, which reflects a reasonable capability of the device to follow a given pressure profile at relatively slow walking speed.

### 3.2. Exosuit Testing Protocol

**Figure 8:**
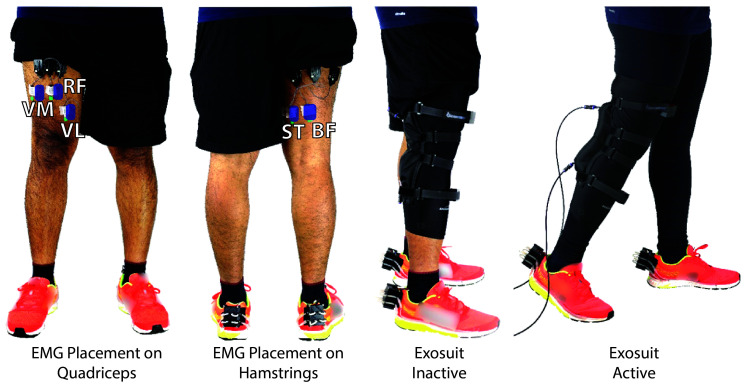
sEMG sensor placement and exosuit inactive and active phases of the test protocol.

To test the controls and the effectiveness of the soft-inflatable exosuit, a human participant protocol is developed. This protocol is applied to three healthy participants of this design study throughout the data collection process, ensuring that all testing phases are executed in the same manner. A written informed consent is obtained from the participants, under an Arizona State University institutional review board approval (PHX-17-0145-70-21). The anthropometric data of the participants is as shown in Table 1. During the test preparation period, the quadriceps and the hamstring region on the thighs are cleaned thoroughly using rubbing alcohol solution. The right regions for placing the surface electromyography sensors (sEMG) (Delsys Trigno${}^{®}$, Delsys, Natick, MA) are identified following the Seniam protocol (Merletti and Hermens, 2000). Five sEMG sensors are placed on the: rectus femoris (RF), vastus lateralis(VL), vastus medials(VF), biceps femoris(BF) and the semitendinosus(ST) muscle groups of the quadriceps and hamstrings, as shown in Figure 8. The maximum voluntary muscle contraction (VMC) and the resting activity of the individual muscle groups is recorded to set the signal limits and offsets.

**Table 1:** Characteristics of the study population.

No.	Gender	Age (years)	Weight (kg)	Height (m)
1	Male	25	65	1.72
2	Male	26	80	1.74
3	Male	25	79	1.65

The walking test procedure of the protocol includes two phases; one, with the exosuit donned, but not active (baseline), and the second with the exosuit donned and active (active). For both phases, the elastic compressing sleeve, where the soft inflatable actuators are mounted on, is worn over the sEMG sensors. In this way, the same uniform pressure is applied to the sEMG sensors during both the baseline and active phases of the study, allowing for fair comparison between the sEMG signals. The baseline is performed with the device already donned on the user, as studies indicate that elastic compression sleeves, such as the one used in the exosuit, are designed to increase proprioception of the joint and not to restrict or enhance physical performance (Faulkner et al., 2013, Venckūnas et al., 2014).

Following all safety requirements, as described in our IRB agreement, the non-impaired adult participants are instructed to walk on an instrumented treadmill with the sEMG sensors attached to the aforementioned muscle groups. The participants walk on the treadmill for three minutes at a slow speed of 0.5 *m/s*, followed by a rest period of five minutes between the baseline and active phases. Each of the test phases is performed three times to ensure accuracy of the collected muscle data (A total of six times for both baseline and device active).

### 3.3. EMG Processing

Following the EMG guidelines suggested by the international society of electrophysiology and kinesiology (ISEK) that enables physiological interpretation of EMG data, the obtained raw sEMG signals from the walking tests are post-processed and filtered using a fourth-order Butterworth filter with a cut-off frequency of 15 Hz. The collected exosuit inactive and active data sets are normalized with respect to the muscle activity during rest and the maximum VMC for each individual muscle group. To compare the sEMG signals from the exosuit inactive and exosuit active tests, five gait cycles from the same time intervals for each set are averaged. These gait cycles are selected from the middle one minute of the total length of each trial. This is performed to ensure that the user gets accustomed to the exosuit in the first one minute and also to eliminate any effects that may arise due to the participant adapting to the external forces exerted by the exosuit.

**Figure 9:**
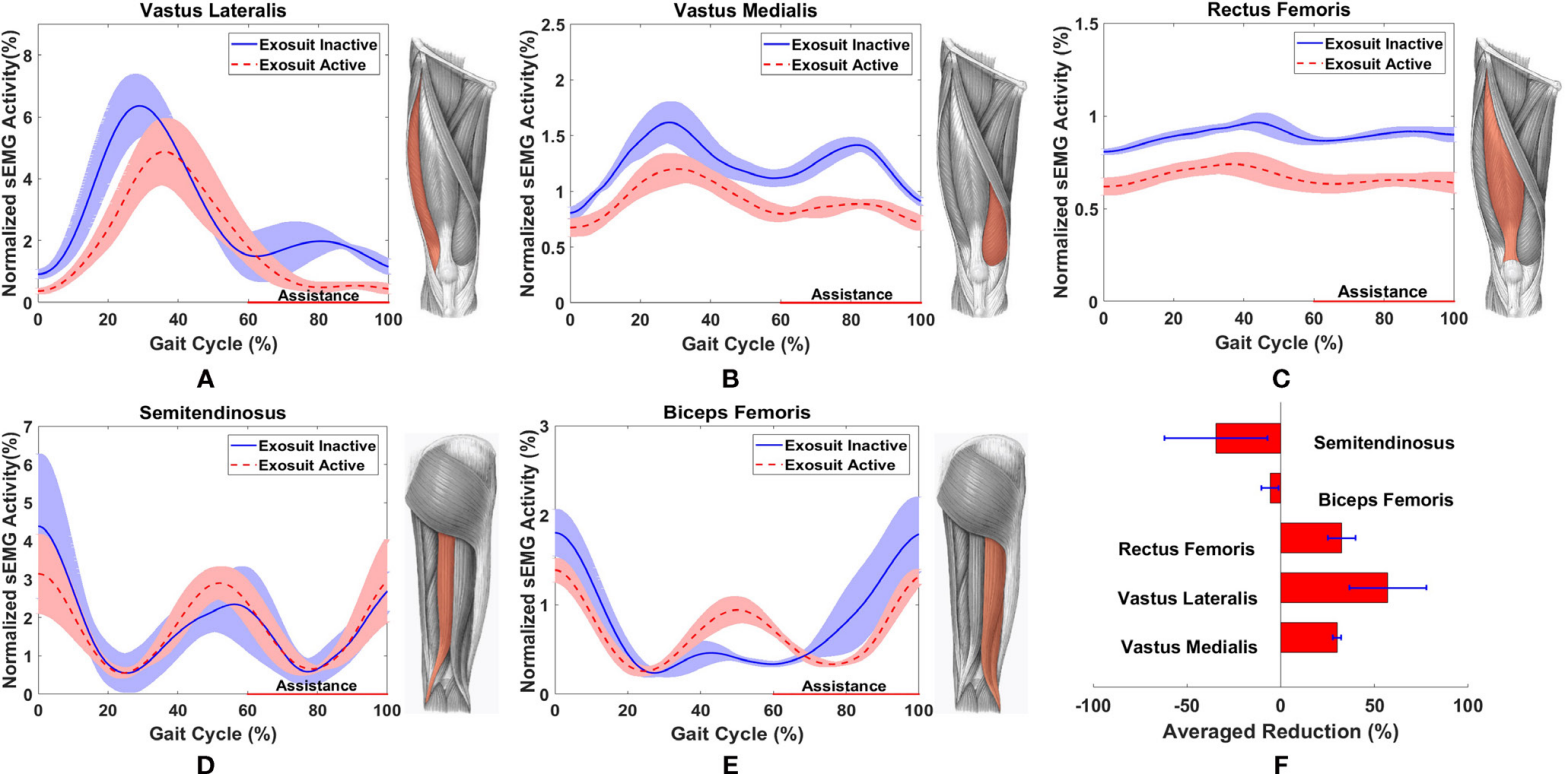
Normalized sEMG signals with reference to seated resting activity and maximum VMC for: **(A)** VL, **(B)** VM, **(C)** ST, **(D)** RF and **(E)** BF averaged for 5 gait cycles for a single test participant. **(F)** Averaged reduction for ST(*n* = 3), BF (*n* = 2), RF (*n* = 2), VL(*n* = 3), VM (*n* = 3) for multiple test participants.

Figure 9A-E, depicts the normalized sEMG activity over the VMC for one test participant for five muscle groups; VL, VM, RF, ST, and BF. Figure 9F, provides the averaged percentage reduction with the SD for the three test participants. An overall reduction of 57.16, 30.06, and 32.5% is observed in the muscle activity of the VL, VM, and RF, respectively, which are the major contributors for the leg extension motion at the knee joint (Snell, 2011).

However, an increase of 5.7 and 34.56% in the BF and ST respectively is also observed when the exosuit is active. The increase in the muscle activity of the hamstrings in the pre-swing phase can be attributed to the delay in deflation leading to resistance in the knee flexion. Additionally, a decrease in the activity of the quadriceps during the stance phase is observed due to the aforementioned latency in deflation which provides an unintended positive assistance to the test participants.

The observed reduction of sEMG activity in the quadriceps during the swing phase when the exosuit is active, supports the set design goals of this work and proves the feasibility of assisting impaired users through a soft inflatable exosuit. It should be noted that the percentage muscle effort is a comparison between the exosuit inactive (baseline) and exosuit active (active) tests, as it is difficult to accurately quantify the individual muscle contributions when the pressure over the sEMG sensors changes due to the compression the elastic sleeve introduces when the exosuit is donned.

## 4. Discussion

In this work, we introduced a two-layer control method for a soft-inflatable exosuit for assisting the knee extension during the swing phase for gait rehabilitation of post-stroke patients. The soft exosuit was integrated with a *smart shoe* system and IMUs for gait phase detection and knee angle measurement respectively, to achieve real-time control. With the gait estimation system added into the soft exosuit system, the exosuit device can provide knee angle information with an RMSE of 0.19*º* and gait event detection in real-time. An actuator stiffness model correlating the stiffness of the actuator to the internal pressure and the bending angle is also generated. The control algorithm is applied to the soft exosuit with a high-level controller utilizing the measurements from the *smart shoe*, the IMUs, and the actuator stiffness model to calculate the desired pressure reference as the controller output. On the other hand, the low-level controller uses the pressure reference output as the control system input to track the pressure using a binary control method and a fluidic pressure sensor to close the feedback loop. Additionally, the lack of a stiffness sensing mechanism for the soft exosuit, makes it difficult to quantify the assistance based only on the knee joint stiffness value. Therefore, to evaluate the performance of the two-layer controller, the low-level pressure controller performance had to be examined more thoroughly. It was observed that the device followed the pressure reference closely during inflation with an overshoot at the peak value while during deflation, the system reacted with a delay attributed to a decaying pressure differential, constant suction rate, and small tubing size. Despite the delay observed during deflation, an RMSE value of 1.27 *kPa* for the pressure tracking experiment was obtained. A reduction in the sEMG activity of the rectus femoris, vastus medialis, and vastus lateralis muscle groups during the gait cycle for three test participants is observed. However, an increase in the muscle activity of the biceps femoris and the semitendinosus muscle groups is also observed as a result of the latency in the deflation of the exosuit. Bearing in mind the design goal of the soft inflatable exosuit, i.e., to assist the knee during extension, the overall positive muscle effort reduction proves the feasibility and leaves room for further exploration on how assistance of multiple muscle groups associated with the knee joint can be accomplished.

## 5. Conclusion

In this paper, a soft-inflatable exosuit designed to provide 25% of the knee moment during the swing phase as well as its integration with IMUs and *smart shoes* insole sensors was presented. Data-driven models for the stiffness of the human knee joint as well as the soft-inflatable actuators were presented. A two-layer stiffness controller for the exosuit was designed and tested with healthy human participants. A significant reduction in the muscle activity of the quadriceps was observed, proving the feasibility of this work.

Future work will include implementation of an improved low-level pressure controller using feed forward algorithms, refining the knee stiffness model by considering the effect of inertia and viscosity term, improving the exhaust rate by adding larger ports and fluid lines, testing of impaired participants to investigate biomechanics and the effectiveness of the suit in them, and finite element modeling of the soft-inflatable actuators.

## Ethics Statement

Under an Arizona State University institutional review board (IRB) approval (PHX-17-0145-70-21), a written informed consent was obtained from healthy participants where they were asked to walk on an instrumented treadmill for a total of six trials with the exosuit active and inactive. 

## Author Contributions

SS and ZQ are equally contributing authors. SS, ZQ, NM, WZ, and PP were involved in the planning and design of this study. SS and PP developed the soft-inflatable exosuit. SS fabricated and assembled the off-board control unit. The control architecture was developed by ZQ and WZ. NM characterized the soft-inflatable actuators. The verification tests were performed by SS and ZQ. The control performance and knee angle data was processed by ZQ and the sEMG data was processed by SS. All authors were involved in writing and formatting the manuscript.

## Conflict of Interest Statement

The authors declare that the research was conducted in the absence of any commercial or financial relationships that could be construed as a potential conflict of interest.
